# Politicization of psychiatry and the improvement of ethical standards in the 1970s

**DOI:** 10.3389/fpsyt.2023.1151048

**Published:** 2023-06-07

**Authors:** Oxana Kosenko, Florian Steger

**Affiliations:** Ulm University, Institute of the History, Philosophy and Ethics of Medicine, Ulm, Germany

**Keywords:** schizophrenia, politicization of psychiatry, forensic psychiatry, ethics, history of psychiatry, code of ethics

## Abstract

**Background:**

The Code of Ethics for Psychiatry adopted by the World Psychiatric Association in 1977, also known as the Declaration of Hawaii, was a milestone in the development of ethical standards in psychiatry. The impetus for the development of the code came primarily from the politicization of psychiatry, first discovered in the USSR, and later in other countries of the socialist camp, such as Romania, Yugoslavia, and the People’s Republic of China. The purpose of this article is to trace reasons for the lack of consolidation among Western psychiatrists against the politicization of psychiatry and their efforts to improve the ethical standards in this medical field.

**Methods:**

We analyzed unpublished documents from the Archive of the German Association for Psychiatry, Psychotherapy and Psychosomatics, the private archives of the West German psychiatrists Gerd Huber and Walter von Baeyer as well as research works. To examine these sources, we implemented the historical-critical method.

**Results:**

The World Psychiatric Association made efforts to collect, analyze and discuss materials concerning psychiatric ethics in order to create the Code of Ethics for Psychiatry and establish an Ethical Committee. In general, the reaction of Western psychiatrists to the information about the internment of dissidents in psychiatric hospitals was restrained and focused on attempts to solve the issue together with the Soviet colleagues.

**Conclusion:**

The international policy of détente of the time as well as collisions between different medical concepts and ethical dimensions did not allow Western psychiatrists to condemn cases of politicization of psychiatry without proir clarification of the situation. The efforts of the World Psychiatric Association in the ethical field improved the ethical standards for psychiatry.

## 1. Introduction

The correlation between the politicization of medicine and dictatorships has been established by medical historians in some research works (1, 2). We would like to point out that the definitions “abuse” or “misuse of medicine” imply the instrumentalization of medicine for political purposes. However, this raises the question about the involvement of physicians in such cases. They shaped medicine and were active actors who did not simply adopt a political system or allow themselves to be instrumentalized (1). Thus, we prefer to use the definition “politicization of psychiatry” that implies not only the political abuse of psychiatry, but also the active participation of psychiatrists themselves in it.

The first Code of Ethics for Psychiatry, known as Declaration of Hawaii, was adopted in 1977 in order to set the ethical standards specific to psychiatry. Among other guidelines, it stated that a psychiatrist must never use the possibilities of the profession for maltreatment of individuals or groups (3). The necessity to create a specific code of ethics for psychiatrists and to fix the mentioned guideline in it was caused by cases of politicization of psychiatry in the USSR in the 1970s. The diagnosis “sluggish schizophrenia,” developed by the leading Soviet psychiatrist Andrei Snezhnevsky, played a key role in the Soviet repressive psychiatry. Dissidents with that diagnosis had to be isolated in psychiatric institutions. The Serbsky All-Union Research Institute of General and Forensic Psychiatry (Serbsky Institute) was the leading one among those institutions, where psychiatric examination of dissidents was conducted. The research on the politicization of psychiatry in the USSR deals in more detail with the period of the 1960s–70s before the 5th Congress of the World Psychiatric Association in Mexico City in 1971, and the period after 1977, when the abuse of psychiatry in the USSR was condemned internationally and the Code of Ethics for Psychiatry was adopted (4–7). It is recognized that the cases of abuse of psychiatry in the Soviet Union prompted the adoption of the Code of Ethics for Psychiatry in 1977 (6). Nevertheless, contemporaries and researchers criticized the World Psychiatric Association and psychiatrists in general for taking too long to condemn the abuses in the Soviet psychiatry (4). They also considered the establishment of an Ethical Committee as a diversionary tactic and the Association’s achievements in the field of ethics as weak (4). However, research papers do not provide a systematic review of the actions taken by the Association and psychiatrists to improve ethical standards in psychiatry. Regarding the reactions of psychiatrists to the politicization of psychiatry in the USSR, medical historians closely scrutinized at the reactions and initiatives coming from British, American, and East German psychiatrists. Therefore, this paper addresses the question why not all Western psychiatrists have immediately condemned the practice of politicizing psychiatry and investigates the efforts they have made to improve the ethical standards in their field. Considering these issues, we focused on the position of Western psychiatrists in general and West German psychiatrists in particular, since the abuse of psychiatry in Nazi Germany was still vivid in the memory of contemporaries.

Our paper is structured as follows. Firstly, we focus on the World Psychiatric Association’s activities to improve ethical standards and create a Code of Ethics for Psychiatry. Then we will discuss the attitude of Western psychiatrists with regard to accusations of Soviet colleagues in the internment of dissidents in psychiatric clinics. In the discussion, we evaluate all the events from the perspective of ethics and its progress in time.

## 2. Materials and methods

To prepare this paper, we analyzed unpublished archival documents and research works. The group of archival documents includes materials that we found in the Gerd Huber Archive, stored at the Bezirkskrankenhaus Günzburg, the Heidelberg University Archives, and the Archive of the German Association for Psychiatry, Psychotherapy and Psychosomatics (Deutsche Gesellschaft für Psychiatrie und Psychotherapie, Psychosomatik und Nervenheilkunde, DGPPN). The documents of a West German psychiatrist Gerd Huber concerning his participation in the conference on schizophrenia in 1973 in the USSR contain his notes as well as his correspondence with the World Psychiatric Association and his colleague Walter von Baeyer. Among von Baeyer’s papers, held in the Heidelberg University Archives, we found his correspondence with the World Psychiatric Association and the minutes of meetings of its Executive Committee. The Archive of the DGPPN contains materials on the abuse of psychiatry in the USSR in the 1970s–80s. In this paper, we have cited almost all of the archival sources that we found in the aforementioned archives. These sources are listed in the references. We have not cite those archival documents that either repeat the content of the cited sources or cover those events that were described in published research works.

We have also evaluated articles of those psychiatrists who participated in the conference on current aspects of schizophrenia in 1973, and research works on the history of politicization of psychiatry in the Soviet Union. To examine these sources, we implemented the historical-critical method, which includes the stages of acquisition of primary sources and research works, critical evaluation of the information contained in primary sources, and presentation of historical data in historical context in terms of objectivity and significance (8).

## 3. Results

### 3.1. The way to the creation of the Code of Ethics for Psychiatry (1977)

Dealing with the cases of politicization of psychiatry in the USSR in the 1970s stimulated debates about ethics among the international psychiatric community. For the first time, the question of forming of an Ethical Committee within the structure of the World Psychiatric Association and adopting a Code of Ethics for Psychiatry came up at the 5th World Congress of Psychiatry in Mexico City in 1971. Although some members of the Association, such as Helmut Ehrhardt (1914–1997), the president of the West German Association for Psychiatry and Neurology, supported the proposal for an Ethical Committee, the idea was not accepted. In May 1972, the World Psychiatric Association circulated the statement of the American Psychiatric Association where it opposed the misuse of psychiatric institutions to detain persons solely based on their political dissent, no matter where it occurred (9). A statement of the West German Association for Psychiatry and Neurology followed (10). However, none of the member societies reacted to those statements. During the Executive Committee of the World Psychiatric Association meeting in Yerevan on October 8th, 1973, a Working Party on ethics was set up. According to Wretmark, the Working Party was created as a compromise after the failed attempt to create an Ethical Committee in 1971 during the 5th World Congress of Psychiatry and under pressure from the American Psychiatric Association (11). The Working Party consisted of Leo Eitinger (1912–1996), a professor of psychiatry at the University of Oslo, Clarence Blomquist (1925–1979), a professor of medical ethics at Karolinska Institutet, and Gerdt Wretmark (1918–2001), a professor of psychiatry at the University of Linköping. The tasks of the Working Party were to identify areas of ethical concern to psychiatrists, to gather information on ethical matters relevant to psychiatrists, and to suggest the aims, functions and composition of an Ethical Committee of the World Psychiatric Association (12). Since the initial suggestion was that the work of the European Commission of Human Rights should be studied first, the Association organized a seminar on human rights held at the European Commission of Human Rights in Strasbourg in 1974. There, ethical concerns in psychiatry and the need for establishing an Ethical Committee within the Association were discussed (13). Soviet psychiatrists Andrei Snezhnevsky (1904–1987) and his colleague Eduard Babayan (1920–2009) attended the meeting, too (14). The seminar showed that instead of establishing a unified worldwide Ethical Committee, it would be more effective to organize seminars at the regional level to discuss ethical issues (15). In 1975, the Association organized seminars on psychiatric ethics, where various ethical codes were discussed. The Hippocratic Oath (470–360 B.C.), the Geneva Declaration (1948), the International Code of Medical Ethics (1949), the Declaration of Helsinki (1964), Sydney (1968), Oslo (1970), and Tokyo (1975) were particularly important. All National Psychiatric Associations with the exception of the one in the USA did not have their own Ethical Codes, only their National Medical Societies’ Codes. Various ethical issues in psychiatry were discussed at the seminar, with a particular focus on the rights of patients. As a result of the seminar, it was suggested to formulate a Declaration of the general principles underlying the ethical practice in psychiatry during the 6th World Congress of Psychiatry in Honolulu in 1977 (16). Since Blomquist was the only university professor of medical ethics in the world at that time, the Association asked him to draft the Code of Ethics in Psychiatry. He evaluated different ethical medical approaches and concluded that while in Europe the paternalistic tradition enshrined in the Hippocratic Oath was strong, in the United States human rights as stated in the Constitution had a greater influence on ethics. Thus, he tried to strike a balance between two approaches and take into account both the patients’ right to self-determination (autonomy), and the protection of their interests by psychiatrists (beneficence) (17). The ethical principles of autonomy and beneficence were stated in the code by formulating the following guidelines: serving the best interests of patients and respecting their self-determination, obtaining an informed consent, confidentiality, an independent proof of compulsory treatment, and the prohibition for abuse of psychiatry. Later, similar codes were adopted at the national level. Moreover, the General Assembly of the World Psychiatric Association during its meeting in Honolulu for the Congress of Psychiatry adopted a resolution in which it condemned the abuse of psychiatry for political purposes in the USSR.

### 3.2. Discussions with Soviet psychiatrists on the internment of dissidents in psychiatric hospitals

Information about the internment of Soviet dissidents in psychiatric hospitals had been circulating among Western psychiatrists since the mid-1960s, but the discussion on this matter began only after the Soviet dissident and publicist Vladimir Bukovsky (1942–2019) smuggled photocopies of forensic reports on prominent dissidents to the West in 1971 (4, 6, 18, 19). Here we need to point out the difference between the notions “dissenter” and “dissident.” The term “dissenter” (*inakomyslyashchiy*) implies that the person to whom it applies “thinks differently.” This refers to moral views that diverge from those generally accepted. The word “dissident” (*dissident*) came into use in the USSR in the 1960s to refer to people who criticized the ideology and authorities of the Soviet Communist Party (20). Thus, dissenters who opposed the political system were called “dissidents.”

During the 5th World Congress of Psychiatry in Mexico City in 1971, psychiatrists were not ready to deliver a statement regarding the internment of Soviet dissidents in psychiatric hospitals until they receive more comprehensive documentation. In 1973, from 8–13 October the World Psychiatric Association held an international symposium on current aspects of schizophrenia in Yerevan and Tbilisi, the capitals of the Soviet republics of Armenia and Georgia. That conference provided an opportunity for a dialogue between Western and Soviet psychiatrists on the issue of dissidents who had been diagnosed with schizophrenia at the Moscow Serbsky Institute. Gerd Huber (1921–1912) and Kurt Heinrich (1925–2015) were invited as speakers from the Federal Republic of Germany. Huber was a professor of psychiatry at the University of Ulm, where he had carried out a big catamnesis study on the course of schizophrenia. He was going to present a paper on the results of his follow-up study of schizophrenia (21). His colleague Heinrich from the University of Düsseldorf was going to give a conference talk on problems of long-term neurolepsy in schizophrenia (22, 23).

Jörg Elten (1927–2017), a reporter of the West German magazine *Stern*, contacted Huber on the eve of the trip and informed him that some Soviet psychiatrists were involved in political campaigns that led to the hospitalization of dissenters in psychiatric hospitals in the USSR (22). Elten just returned from Moscow, where he had interviewed Andrei Sakharov (1921–1989), a Soviet nuclear physicist, dissident and activist for human rights (24). The latter wanted to know Huber’s and Heinrich’s views and how they would behave towards their Soviet colleagues during the symposium (22). Huber contacted the Secretary General of the World Psychiatric Association Denis Leigh (1915–1998) and asked whether Leigh could encourage the Western participants of the symposium to appeal to Soviet colleagues and ask them to permit an international commission of psychiatrists to examine the dissenters declared mentally ill (25). “Heinrich and I are now in a particularly precarious position, since psychiatry has already been misused for political purposes in Nazi Germany,” noted Huber his distress (25). Leigh rejected a proposal on the grounds that no national psychiatric society complained about the Soviet one and he would not pressurize anyone from the Western participants (26).

As the internment of dissidents in Soviet psychiatric institutions was discussed in the Western press, the All-Union Society of Neuropathologists and Psychiatrists decided to invite conference participants to meet at the Serbsky Institute in order to discuss the allegations about the Soviet forensic psychiatry (4, 27). The World Federation of Mental Health adopted a resolution, in which it welcomed the invitation of the Soviet Health Ministry to conference participants for an independent examination of cases where a wrongful certification had been alleged (12). During the meeting of the Executive Committee of the World Psychiatric Association, its members decided to attend the Serbsky Institute on October 15th, 1973. However, it was resolved that they would go there as observers without expressing any personal opinions and make a committee decision together.

A group of conference participants, Huber and Heinrich included, agreed to accept the invitation, but specified in advance that such a complex issue could not be resolved in one day. “It is therefore unlikely that a clear-cut statement of approval or disapproval will be made at the end of the meeting,” their statement declared (28). However, upon arrival in Moscow some participants, including Huber and Heinrich, forwent their original intention (29). In the end, 13 participants from Western countries attended the meeting (4, 30, 31). They evaluated six medical records of prominent dissidents, who were diagnosed by the Soviet psychiatrists as mentally ill, and took part in the examination of one dissident as a “typical case” for the whole group. After that meeting, the Soviet propaganda spread rumors in the USSR that Western psychiatrists had confirmed the diagnosis of schizophrenia for the dissidents (4). However, the meeting participants did not draw unequivocal conclusions and viewed the meeting as the beginning of a discussion that would stimulate further communication between Soviet and Western colleagues (30).

Upon the return of the speakers from the conference, a discussion broke out on the pages of the *British Medical Journal* laid down by Wing’s article *Psychiatry in the Soviet Union* published in March 1974 (30). John Wing (1923–2010) was a professor of social psychiatry at the Institute of Psychiatry in London and was internationally known for his studies in the field of schizophrenia (32). His article dealt with the concept of schizophrenia, the responsibility of patients for their actions, and politicization of psychiatry. Wing emphasized three differences between the forensic psychiatry in the USSR and in the Western countries, which made the politicization of psychiatry possible in the USSR. First of all, it was a political distinction, stated in the fact that for Soviet citizens to express an opinion against the political course was a crime against their country. Such citizens would not be considered criminals in Western countries where freedom of speech was respected. Further, Wing pointed out that the Soviet concept of schizophrenia differed from the one shared by British psychiatrists, which he would rather characterize as a personality disorder. As for those Soviet dissidents who were locked up in psychiatric hospitals only because they had developed complex economic and social theories alternative to Marxism, he would not diagnose such persons with schizophrenia or any mental disorder. As for those Soviet dissidents who have been locked up in psychiatric hospitals only because they have developed complex economic and social theories alternative to Marxism, he would not have diagnosed such persons with schizophrenia or any mental disorder (30). Finally, it seemed ludicrous to Wing that a person who was not seriously ill by Western medical standards should be responsible for actions that were not considered crimes in Western democracies. But he suggested that the question of responsibility was different for the Soviet psychiatrists: “Is a person who is suffering from a slowly developing form of schizophrenia responsible for an action which is likely to land him, at the very least, in a labour camp for 3 years?” (30).

The director of the Serbsky Institute Georgi Morozov (1920–2012), countered that the concept of schizophrenia in the USSR was similar to that shared by Western scientists (31). It must be noted here, that he was considered one of the leading psychiatrists in the country. His authority in the Soviet professional community especially strengthened after 1975. In 1969 he was elected a corresponding member and in 1975 a full member of the USSR Academy of Medical Sciences. From 1975 to 1988 Morozov was the chairman of the board of the All-Union Scientific Society of Neuropathologists and Psychiatrists (33).

The West German psychiatrist Walter Ritter von Baeyer (1904–1987) entered the discussion and tried to move it to the plane of purely psychiatric analysis of the problem. Von Baeyer was a professor of psychiatry at the University of Heidelberg from 1955 to 1972 and a former vice president of the World Psychiatric Association from 1966 to 1971 (34). He was an active advocate for patients and human rights, and in 1977, he co-founded the German Association against Political Abuse of Psychiatry (DVpMP), which has borne the name Walter-von-Baeyer-Gesellschaft für Ethik in der Psychiatrie e. V. (GEP) since 1999. On the example of the Soviet dissident Peter Grigorenko (1907–1987), von Baeyer showed the groundlessness of the diagnosis of schizophrenia in his case. Analyzing the case of Grigorenko, whose „reformist ideas” were regarded by Soviet psychiatrists as pathological deviations, he concluded that Grigorenko’s views could not be assessed as “absurd, bizarre, or egocentric” as it would be the case, for example, in a paranoid schizophrenic delusion (35). Finally, he urged psychiatrists and their professional communities to take a critical view of the problem and denounce the abuse of psychiatry in the USSR in order to prevent further misuse of forensic psychiatry. The Executive Committee of the World Psychiatric Association decided during its meeting held on November, 10, 1974 to send an appropriate denial to Morozov’s statement to the *British Medical Journal.* That happened despite the dissent of the USSR representative in the Executive Committee, Associate Secretary Marat Vartanyan (1932–1993), who insisted that the letters of individuals should not necessarily require an answer (36). Thus, the Secretary General of the World Psychiatric Association Leigh put an end to the discussion of the results of the conference in the USSR in his article from 1975 (36). He distanced the Association from the results of the meeting of conference participants at the Serbsky Institute, stressing that they had attended the meeting in a personal, non-official capacity. He tried to defend the Association against accusations of being ambiguous and instead emphasized the competence of the European Commission of Human Rights with regard to the situation of dissidents (37).

## 4. Discussion

The research works suggest some reasons for the lack of consolidation among Western psychiatrists against the politicization of psychiatry in the USSR in the 1970s. The researchers rightly point to the international policy of détente, which made cooperation and experience exchange between Soviet and Western psychiatrists possible (4, 19). Therefore, the condemnation of Soviet colleagues and their expulsion from the World Psychiatric Association would have led to the end of cooperation and the closure of research programs. The presence of a political component also confirms the fact that the formation of the Working Party on ethics in 1971 from the “neutral” Scandinavians was done intentionally, so it could be approved by national associations of both Western and Eastern countries (11). In addition, engagement among psychiatrists depended on whether they trusted the evidence that was offered as a proof of indictment of Soviet colleagues. As for the Ethical Committee, some researchers believe that Leigh’s proposal to form it was merely a diversionary tactic, because the committee was supposed to deal with formulating general ethical principles, not handling specific issues (4). We must also point out the collisions between different medical concepts and ethical dimensions, while evaluating attempts of Western psychiatrists to clarify the situation of politicizing psychiatry in the USSR, and to improve the ethical standards in their field.

### 4.1. Medical concepts of schizophrenia

One of the stumbling blocks in the discussion of the politicization of medicine in the USSR was the discrepancy in the medical concepts of Western and Soviet psychiatrists. First of all, it concerned the concept of schizophrenia. Although during the discussion on pages of *British Medical Journal*, Morozov countered that the concept of schizophrenia in the USSR was similar to that shared by Western psychiatrists, there was still a big difference. During the 1960s and 1970s, Western psychiatrists progressively narrowed the concept of schizophrenia, limiting the diagnosis to the most severe forms. In contrast, the Moscow school of Snezhnevsky defined the concept of schizophrenia broadly including both clinical schizophrenic manifestations and latent non-psychotic forms (residual mental disorder) (4). Moreover, Snezhnevsky introduced the concept of the so called “sluggish schizophrenia” – a type of schizophrenia in which the disease progresses weakly without the productive symptomatology common to schizophrenic psychoses, but most often with only indirect clinical manifestations and minor personality changes. That broad interpretation of schizophrenia allowed Soviet psychiatrists to declare people who expressed criticism of the state political system legally incompetent.

### 4.2. Ethical dimensions

Western psychiatrists assumed that there were certain cases of abuse in psychiatry in every country. Therefore, they treated the first news about the internment of Soviet dissidents in psychiatric hospitals not as a widespread practice, but as isolated cases. As it appears from the internal documents of the Association, the exclusion of Soviet psychiatrists was not ruled out. However, the condemnation of Soviet psychiatrists and their exclusion from the World Association could not solve the issue as such. Western psychiatrists urged a dialogue with their Soviet colleagues and called for an international commission that could evaluate the dissidents. Particularly under the pressure from the Western public opinion, the All-Union Society of Neuropathologists and Psychiatrists decided to invite the participants of the 1973 symposium to a discussion. Since Western psychiatrists were solution-oriented, their Soviet colleagues continued to participate in seminars on ethics organized by the Association.

As for the establishment of the Ethical Committee, it should be noted that medical ethics was not yet established as a discipline at that time, and the formation of an Ethical Committee was not an easy task. Here we must disagree with those researchers who treated the establishment of an Ethical Committee as a diversionary tactic and the Association’s achievements in the field of ethics as weak (4). It seems to us that the activities organized by the Association in the field of ethics were important and eventually led to the creation of the Code of Ethics for Psychiatry. The code not only prohibited the abuse of psychiatry, but also formulated important ethical principles of a patient’s autonomy and beneficence. Together with the principles of non-maleficence and of justice, formulated by Beauchamp and Childress a year later (38), they constituted four principles of biomedical ethics that are still guiding physicians today.

Other ethical aspects played an important role, too. The members of the Executive Committee of the World Psychiatric Association were particularly cautious about signing documents whose content was directed against one of its member societies. In 1971, before the 5th World Congress of Psychiatry, the Executive Committee did not make any statement because the Soviet Association of Psychiatry had not even had the opportunity to comment on all accusations. They knew only one side of the coin, and considered it their duty to listen to the other side as well before taking a position.

Moreover, the memory of World War II and the abuse of psychiatry in Nazi Germany was an important factor for German psychiatrists. Thus, von Baeyer held the view that German psychiatrists had no right to be the first ones to condemn their Soviet colleagues precisely because of the Nazi past. Huber and Heinrich held the same views. Perhaps that is why they refused to accept the invitation of the Serbsky Institute.

## 5. Conclusion

Analyzing archival materials and research concerning the practice of politicization of psychiatry in the USSR, we conclude that Western psychiatrists were oriented toward a mutual clarification of the issue with their Soviet colleagues. The World Psychiatric Association’s efforts to collect, analyze and discuss materials concerning psychiatric ethics, to create the Code of Ethics and the Ethical Committee cannot be qualified as weak. We argued that not only an international policy of détente of the time, but also collisions between different medical concepts and ethical dimensions did not allow Western psychiatrists to condemn politicization of psychiatry in the USSR without clarifying the situation. Only after receiving increasing evidence regarding internment of healthy dissidents in psychiatric hospitals, neglect by Soviet colleagues to jointly address the issue, and psychiatric evaluation of dissidents who had immigrated to Western countries, the practice of politicizing psychiatry in the USSR became evident, and Soviet psychiatrists were forced to leave the World Psychiatric Association in 1982. The main issue in dealing with allegations against Soviet forensic physicians was that Western psychiatrists received only limited knowledge and could not access reliable information. Thus, we learned that in such a difficult situation physicians must always be aware of context sensitivity and all backgrounds when making decisions. Discussions about politicization of psychiatry in the USSR not only initiated the creation of the Code of Ethics for Psychiatry. They showed how important ethics and the further development of ethical principles are. Human rights violations by mental health services have led to the creation of international and national initiatives on psychiatry. Ethical debates in psychiatry have sensitized physicians to the issue of patients’ rights as well as ethical principles, which over time have become an indispensable part of a physician’s daily routine.

## Data availability statement

The original contributions presented in the study are included in the article/supplementary material, further inquiries can be directed to the corresponding author.

## Author contributions

OK and FS conceptualized and designed the study. OK collected the sources and wrote the first draft of the manuscript. OK and FS analyzed the sources as well as contributed to the manuscript revision, read, and approved the submitted version.

## Conflict of interest

The authors declare that the research was conducted in the absence of any commercial or financial relationships that could be construed as a potential conflict of interest.

## Publisher’s note

All claims expressed in this article are solely those of the authors and do not necessarily represent those of their affiliated organizations, or those of the publisher, the editors and the reviewers. Any product that may be evaluated in this article, or claim that may be made by its manufacturer, is not guaranteed or endorsed by the publisher.
